# Transesophageal Echocardiography vs. Transthoracic Echocardiography for Methicillin-Sensitive Staphylococcus aureus and Methicillin-Resistant Staphylococcus aureus Endocarditis

**DOI:** 10.7759/cureus.39996

**Published:** 2023-06-05

**Authors:** Elvis Henriquez, Neha Fatima, Rithika Sayabugari, Muhammad Hamza Nasim, Hafseena Noorayingarath, Karoona Bai, Alberto Garcia, Ayesha Habib, Tirth P Patel, Fouziya Shaikh, Waleed Razzaq, Zain U Abdin, Ishita Gupta

**Affiliations:** 1 Internal Medicine, University of Medical Sciences, Las Tunas, CUB; 2 Internal Medicine, Lisie Hospital, Kochi, IND; 3 General Medicine, Gandhi Medical College, Hyderabad, IND; 4 Internal Medicine, Lahore Medical and Dental College, Lahore, PAK; 5 Internal Medicine, Kanachur Institute of Medical Sciences, Natekal, IND; 6 Internal Medicine, Dow University of Health Sciences, Civil Hospital Karachi, Karachi, PAK; 7 Internal Medicine, Universidad Javeriana, Bogota, COL; 8 Internal Medicine, Punjab Medical College, Faisalabad, PAK; 9 Medicine, S.S. Hospital, Petlad, IND; 10 Internal Medicine, Krishna Institute of Medical Sciences, Karad, IND; 11 Internal Medicine, Services Hospital Lahore, Lahore, PAK; 12 Medicine, District Head Quarters Hospital, Faisalabad, PAK; 13 Medicine, Dr. Rajendra Prasad Government Medical College, Kangra, IND

**Keywords:** infective endocarditis, methicillin-resistant staphylococcus aureus, methicillin-sensitive staphylococcus aureus, transthoracic echocardiography, transesophageal echocardiography, positron emission tomography-computed tomography scan, radiolabeled leukocyte scintigraphy, duke criteria, meca gene

## Abstract

Infective endocarditis is an infection of the inner layers of the heart, seen often in intravenous drug users and patients with valvular lesions or prosthetic heart valves. This entity has high mortality and morbidity. The most common causative microorganism is *Staphylococcus aureus*. In this comprehensive literature review, we focused on both *Staphylococcus aureus* infections, i.e., methicillin-sensitive *Staphylococcus aureus* (MSSA) and methicillin-resistant *Staphylococcus aureus* (MRSA) endocarditis, demographics, use of transthoracic echocardiogram and/or transesophageal echocardiogram for diagnostics, and treatments. Although clinical criteria are relevant, transesophageal echocardiogram plays a vital role in establishing and identifying the presence of infective endocarditis and its local complications, with higher sensitivity in patients with prosthetic valves. The antibiotic selection posed a great challenge for clinicians due to antibiotic resistance and the aggressive nature of *Staphylococcus aureus*. Early diagnosis of infective endocarditis, when suspected, and effective management by a multispecialty team can improve the outcome for the patients.

## Introduction and background

Endocarditis

Infective endocarditis (IE) is defined as an infection of the inner layer, endothelium, of the heart or an implantable heart device, which may include prosthetic heart valves [1]. Even with the advancement in medicine, the mortality rate due to IE remains as high as 30% in one year [1].

The most common organism involved in causing IE is *Staphylococcus aureus* [2]. Other causative organisms include the HACEK organisms (*Haemophilus parainfluenzae*, *Aggregatibacter actinomycetemcomitans*, *Cardiobacterium hominis*, *Eikenella corrodens*, and *Kingella kingae*), which are also a part of the normal flora of the mouth [3]. IE diagnosis is based on the modified Duke's criteria, which involve clinical presentation, lab results, and echocardiography [4]. However, with advancements in medical science, updates have been made to Duke's criteria, and a new 2023 Duke-ISCVID criteria have been proposed by the International Society for Cardiovascular Infectious Diseases (ISCVID). This criterion involves using newer microbiological, imaging, and intraoperative techniques to diagnose IE [5]. Initially, blood cultures and echocardiography are done. Echocardiography is used to observe the number of vegetation, its size, and its location on heart surfaces [6]. Other than that, CT scans, MRIs, and nuclear imaging can also be used to diagnose IE [7].

As antibiotic resistance is increasing, newer antibiotics have been used in combination with older regimens for effective control [7]. Antibiotics used in the treatment of IE include aminoglycosides and penicillin. Alternatives for methicillin-sensitive bacteria include cefazolin and vancomycin, and daptomycin can be used for methicillin-resistant bacteria. Patients having prosthetic valves are given gentamicin in addition to penicillin and aminoglycosides. This treatment is continued for prosthetic valve endocarditis for four to six weeks [8]. Antibiotic therapy is preferably given intravenously, as giving oral antibiotics has no added benefits [9]. IE is a high-mortality disease and requires a team of physicians with different expertise to treat it [10,11]. Most frequent complications include valve regurgitation and heart failure. The vegetation can embolize and affect the brain, spleen, kidneys, and lungs, where they cause infarction [12].

Methicillin-sensitive *Staphylococcus aureus* and methicillin-resistant *Staphylococcus aureus*

One of the causes of hospital and community-acquired bacteremia is *Staphylococcus aureus*. Methicillin-sensitive *Staphylococcus aureus* (MSSA) and methicillin-resistant *Staphylococcus aureus* (MRSA) are two different forms of *Staphylococcus* infections that can be distinguished by how they respond to treatment. An oxacillin minimum inhibitory concentration (MIC) of greater than or equal to 4 micrograms/ml indicates that *S. aureus* is resistant to penicillins [13].

One of the most common causes of hospital-acquired infections is MRSA, which is frequently linked to severe morbidity, death, length of stay, and financial burden. The skin and subcutaneous tissues constitute the most frequently infected organs by MRSA, followed by invasive infections like osteomyelitis, meningitis, pneumonia, endocarditis, lung abscess, and empyema [13]. Complications can be extremely severe with chances of recurrence, which can make treatment difficult [14]. The mecA gene sequence, known to produce transpeptidase PB2a and reduce the organism's affinity to bind to beta-lactam antibiotics, is the primary cause of MRSA resistance to these drugs [15].

Due to many factors, such as different local infection management practices and pathogen-specific traits of the circulating clones, there is considerable geographic variability in the MRSA burden [16]. The prevalence of MRSA in Scandinavian countries is 1% to <5%, whereas prevalence in parts of America and Asia ranges from 25% to ≥ 50%. Prevalence in the Australian continent ranges from 10% to <25%. According to statistics from the majority of nations, the prevalence of MRSA has been rising since the early 2000s, while it has started to fall in South Africa from 36% in 2006 to 24% between 2007 and 2011 [16].

Transthoracic echocardiography

Transthoracic echocardiography (TTE) remains a critical instrument in clinical cardiology. The procedure frequently aids as one of the primary tomography modalities in assessing cardiac illness due to low-priced, movability, extensive availability, shortage of ionizing radiation, and capability to assess both anatomy and function of the heart. Therefore, most patients who go through a cardiac computed tomography (CT) or magnetic resonance (MR) tomography examination will have a TTE accessible for examination. Subsequently, it exists imperious that cardiac imagers be acquainted with the essentials of a routine TTE examination and communal TTE drawbacks and limitations that might lead to a recommendation for cardiac CT or MR imaging. Known difficulties and constraints of TTE will be emphasized by utilizing cardiac CT and MR imaging as the problem-resolution modality [17].

Some of the limitations of TTE include the following: (i) masses and mass mimics (crista terminalis, eustachian valve, right ventricle septomarginal trabecula, coronary sulcus fat, left ventricular band (or left ventricular false tendon), hiatus hernia, caseous calcification of the bicuspid valve, lipomatous hypertrophy of the interatrial septum, heart tumors); (ii) unwell envisioned apical lesions (aneurysm, thrombus, infarction, hypertrophic and supplementary nonischemic cardiomyopathies); (iii) evaluation for rising thoracic aortic divisions (false positive, false negative, dissecting aneurysms); (iv) pericardial illness (acute and chronic/constrictive pericarditis, pericardial tamponade, pericardial cysts, and diverticula, congenital nonexistence of the pericardium) [17].

Another form of TTE is a bubble contrast TTE. In this procedure, saline is injected into a vein, and then the echocardiography study is performed. The bubbles are initially spotted in the right atrium and right ventricle. If bubbles are seen in the left heart, it might suggest a shunt, such as a patent foramen ovale, atrial septal defect, ventricular septal defect, or arteriovenous malformations in the lungs [18].

Transesophageal echocardiography

Transesophageal echocardiography (TEE) is a medical imaging technique used for producing better and more detailed images of the heart to delineate minute pathology often missed by traditional echocardiography. TEE uses a probe that is inserted into the esophagus, which gets close to the heart. TEE is considered to be a slightly invasive procedure.

The TEE probe contains a small ultrasound transducer that emits sound waves and receives the echoes that bounce back from the heart. These echoes are then used to create a detailed image of the heart's structures, including the chambers, valves, and blood vessels [19].

TEE is often used in patients who cannot undergo traditional echocardiography or in cases where a more detailed evaluation of the heart is required. It is particularly useful in evaluating (a) the function of the heart valves, (b) identifying blood clots, (c) assessing the overall function of the heart, and (d) thoracic aortic dissection, pulmonary embolism, and endocarditis [20].

TEE is generally considered a safe procedure, but there is a small risk of complications, such as bleeding, infection, or damage to the esophagus [19]. Overall, TEE is a valuable diagnostic tool in the evaluation and management of various cardiac conditions [19].

Our review

The literature comparison of TTE and TEE for IE caused by MRSA and MSSA highlights several issues. It calls into question the surgery's timing and whether it impacts the patient's prognosis. Additionally, the diagnostic window for patients with prosthetic implants, cardiac implantable electronic devices (CIEDs), or valve replacements needs to be expanded to make an appropriate diagnosis of IE. Besides, it is essential to determine which patient, procedure, and device-related risk factors put patients with valve replacements, prosthetic implants, or CIEDs at a greater risk of IE. If TTE and TEE are inconclusive but suspicion is still high, another concern is whether multimodality imaging and molecular biology procedures should be included in the guidelines. If these alternative diagnostic techniques are often used, one must also take into account their cost-effectiveness and the possibility that they are only accessible in specific centers. If both TTE and TEE are negative, when should they be repeated? Alternatively, should TEE be employed as a first-line imaging modality for individuals with a considerably higher risk of IE? Another thing to think about is whether imaging techniques like TTE/TEE should be used during postoperative care or when patients are being monitored after starting antibiotic therapy.

## Review

Methodology

Search Method and Strategy

We conducted a comprehensive literature review to find relevant articles by searching through the PubMed database from April 1st, 2023 to April 15th, 2023. This task was done by two authors from our team. Relevant keywords and terms used for the search strategy included “MSSA,” “methicillin-sensitive *Staphylococcus aureus*,” “methicillin-resistant *Staphylococcus aureus*,” “endocarditis,” “MRSA,” “transthoracic echocardiogram,” “transesophageal echocardiogram,” “TTE,” and “TEE.” Then, the above keywords were used to find relevant research articles. We used the snowballing technique to include articles. Our search included publications from a decade (2012-2023) and case reports that reported adult patients. After a thorough review of each article, we organized each publication using a data extraction sheet that included the following sections: “duration of hospital stay,” “the number of participants,” “age,” “chief complaint,” “sex,” “comorbidities,” “ presence of MRSA or MSSA,” “when was MSSA/MRSA confirmed,” “was TTE done before/during/after TEE,” “type of endocarditis/vegetation was present on TTE,” “when was TEE used,” “treatment,” and “outcome.” All conflicts were resolved via discussion with a senior author.

Data Screening and Eligibility

The inclusion and exclusion criteria of our review have been illustrated in Table 1 below.

**Table 1:** Inclusion and exclusion criteria TTE: transthoracic echocardiogram; TEE: transesophageal echocardiogram.

Inclusion criteria	Exclusion criteria
Published case reports with PubMed indexing	Observational studies, systematic reviews, meta-analyses, case series, randomized controlled trials (open-labeled, double-blinded, and triple-blinded)
Studies that included only human data	Studies conducted on pediatric patients
Articles in the English language only	Articles in languages other than English
Studies that included adult patients	Studies where TTE and TEE were not carried out
Studies that included patients who were diagnosed with infective endocarditis	Articles that were not peer-reviewed
Studies involving patients with positive blood cultures for *Staphylococcus aureus*	In vitro studies
Studies in which TTE and TEE were performed on patients to look for vegetation	

Thereby, we had a total of 10 case reports [21-30], which we included in our study. All the patients included in these studies had blood cultures positive for either MSSA or MRSA. They are listed in Table 2.

**Table 2:** Records used in our review

Title	Authors
Acute endocarditis in intravenous drug users: a case report and literature review [21]	Yan Ji, Lara Kujtan, Dawn Kershner
The value of screening for bicuspid aortic valve in first degree family members [22]	Emad Kanda, Atefeh Kalantary, Nouraldeen Manasrah, et al.
A large mitral valve vegetation not visualized on transthoracic echocardiography: A case report [23]	Nabil Braiteh, Kareem Ebeid, Alon Yarkoni, et al.
Penile implant infection resulting in *Staphylococcus aureus* bacteraemia and infective endocarditis [24]	Joseph P Creel, David Triplett, Mannu Nayyar, et al.
Tricuspid valve endocarditis: A disguise in multifocal septic arthritis [25]	Zain U Abideen, Rehman M Bhatti, Farhan Khalid, et al.
Transcatheter aortic valve replacement associated infective endocarditis case series: broadening the criteria for diagnosis is the need of the hour [26]	Kriti Lnu, Shamim Ansari, Shantanu Mahto, et al.
*Staphylococcus aureus* infective endocarditis [27]	Julia Grapsa, Christopher Blauth, YS Chandrashekhar, et al.
A case of rapid development of methicillin‑resistant *Staphylococcus aureus* mechanical aortic root abscess despite appropriate antibiotic use [28]	Sanjay Chandrasekhar, Dae Hyun Lee, Nidhi Patel, et al.
Methicillin resistant *Staphylococcus aureus* infective endocarditis presenting as neutrophilic meningoencephalitis [29]	Tushar Bajaj, Anthony Karapetians, Natalie Karapetians, et al.
Unmasking severe tricuspid valve regurgitation after percutaneous debulking of large tricuspid vegetation [30]	Adrian Mercado-Alamo, Hemindermeet Singh, Howard Rosman, et al.

Data Collection and Analysis

Once the articles were finalized, we extracted data from all the studies. The studies were all compiled in one place and sorted according to the first author and DOI. Data were collected in the following categories when available: demographic features like age and sex; duration of hospital stay; the number of participants; chief complaint; comorbidities; kind of bacteria (MRSA vs. MSSA); the day of diagnosis; diagnostic tests, i.e., TTE vs. TEE; findings on echocardiogram; treatment; and outcome.

We tabulated the data using Microsoft Excel (Microsoft Corporation, Redmond, WA). Referencing was done according to guidelines using EndNote (Clarivate, London, UK). This study did not require ethical approval as data were obtained from already available databases and patients were not directly involved. A summary of the data has been presented in Table 3.

**Table 3:** Summary of data extracted Afib: atrial fibrillation; AVR: aortic valve replacement; CABG: coronary artery bypass grafting; CAD: coronary artery disease; DM: diabetes mellitus; GERD: gastroesophageal reflux disease; HTN: hypertension; IV: intravenous; OSA: obstructive sleep apnea; T2DM: type 2 diabetes mellitus; TAVR: transcutaneous aortic valve replacement; TEE: transesophageal echocardiogram; TTE: transthoracic echocardiogram.

Authors	Age	Sex	Comorbidities	Chief complaint	Was TTE done before TEE	Signs of endocarditis/vegetations on TTE	When was TEE done	Signs of endocarditis/vegetation on TEE	Treatment	Outcome
Emad Kanda, Atefeh Kalantary, Nouraldeen Manasrah, et al. [22]	54 y/o	Male	HTN, T2DM, GERD, OSA	Fever, cough, dyspnea, pedal edema	Yes (before)	None		1.4 cm mobile vegetation on the aortic valve, an abscess on the aortic annulus	AVR, root abscess repair, ascending aortoplasty, CABG, antibiotics for eight weeks	Successfully recovered
Sanjay Chandrasekhar, Dae Hyun Lee, Nidhi Patel, et al. [28]	74 y/o	Male	Mechanical aortic valve replacement, DM, obesity	Fever, abdominal pain	Yes (before)	None	First TEE done on the same day, repeat TEE after 5 days	Thickening and echo-lucency posterior to aortic valve	Vancomycin, aztreonam, metronidazole	Passed away
Zain U Abideen, Rehman M Bhatti, Farhan Khalid, et al. [25]	69 y/o	Female	Hypothyroidism, anal carcinoma	Fever, joint pain (septic arthritis), myalgia	Yes (before)	None		Tricuspid valve vegetation	Tricuspid valve repair with annuloplasty and replacement of septal leaflet with the pericardium, nafcillin for 6 weeks	Successfully recovered
Yan Ji, Lara Kujtan, Dawn Kershner [21]	45 y/o	Male	IV drug user, HTN, T2DM, hepatitis C	Altered mental status	Yes (before)	None	5 days after admission	1.5 cm aortic valve vegetation	Nafcillin and gentamicin	Cardiac arrest and died
Julia Grapsa, Christopher Blauth, YS Chandrashekhar, et al. [27]	19 y/o	Female		Fever, abdominal pain, myalgia	Yes (before)	Vegetations on the mitral valve and right ventricular valve	On the same day (intraoperatively)	Vegetation on mitral and tricuspid valves and papillary muscle	Mitral and tricuspid valve repair, flucloxacillin	Successfully recovered
Nabil Braiteh, Kareem Ebeid, Alon Yarkoni, et al. [23]	41 y/o	Male	IV drug user, chronic hepatitis C	Fever, altered mental status, myalgia	Yes (before)	None		30 x 30 cm vegetation on the anterior mitral valve leaflet	Mitral valve replacement, ceftriaxone and vancomycin	Successfully recovered
Adrian Mercado-Alamo, Hemindermeet Singh, Howard Rosman, et al. [30]	27 y/o	Male	Polysubstance abuse including IV drug use, hepatitis C	Malaise, fatigue	Yes (before)	2.6 cm tricuspid valve vegetation	On the same day	Tricuspid valve vegetation	Angiovac venous drainage system	Severe tricuspid regurgitation
Joseph P Creel, David Triplett, Mannu Nayyar, et al. [24]	50+ y/o	Male	HTN, DM, inflatable penile prosthesis	Fever, scrotal pain	Yes (before)	None		1.1 x 0.7 cm vegetation on anterior mitral valve leaflet	Explant of the prosthesis, cefazolin IV for 6 weeks	Successfully recovered
Tushar Bajaj, Anthony Karapetians, Natalie Karapetians, et al. [29]	53 y/o	Female	Pacemaker, hip replacement hardware	Fever, low back pain	Yes (before)	None		0.8 cm x 0.3 cm vegetation on atrial pacer	Vancomycin and ceftriaxone	Successfully recovered
Kriti Lnu, Shamim Ansari, Shantanu Mahto, et al. [26]	79 y/o	Male	TAVR, HTN, T2DM	Altered mental status, back pain	Yes (before)	None		No vegetation, thickening in the posterior aspect of the aortic root	Surgical exploration, antibiotics	Successfully recovered
Kriti Lnu, Shamim Ansari, Shantanu Mahto, et al. [26]	71 y/o	Male	TAVR, HTN, CAD, paroxysmal afib	Fever, altered mental status, abdominal pain	Yes (before)	None		None	Surgical exploration, antibiotics	Comfort care

Results

Demographics

Our review covered 10 case reports for a total of 11 patients [21-30]. There were only three females [25,27,29] accounting for 27% and eight males accounting for 73% [21-24,26,28,30].

The age range was 19-79 years with an average age of 52.9 years. Although not all the cases reported hospitalization stays, the average in-patient stay was 23.3 days.

It is relevant to mention that two patients had histories of aortic valve replacement [28], pacemaker, and hip prosthetic implantation [29]. Despite receiving antibiotic treatment, the first patient who had undergone aortic valve replacement seven years prior developed an aortic root abscess. The blood cultures revealed that the infection was caused by MRSA, highlighting the aggressiveness of this microbe and its association with aortic root abscess [28]. The other patient developed IE related to their intracardiac device implantation. She had previously developed a superficial skin infection, which later allowed direct access to the heart by MRSA [29].

The other comorbidity cases were attributed to hypertension with five patients [21,22,24,26] and type 2 diabetes mellitus also with five patients [21,22,24,26,28]. The three patients diagnosed with chronic hepatitis C have also reported a history of intravenous drug use [22,23,30]. We also noted two patients with hyperlipidemia, one patient with hypothyroidism, and one patient who was recently diagnosed with anal carcinoma. End-stage chronic kidney disease, stroke, obstructive coronary artery disease, atrial fibrillation, osteomyelitis, schizoaffective disorder, and tobacco with polysubstance abuse were each noted in one patient.

The cultures performed in these patients resulted in eight MSSA [21-27], and three MRSA [28-30].

When and How Was MSSA or MRSA Found?

Blood cultures were performed on all 11 patients, and all yielded a positive result for either MRSA or MSSA. The species were isolated in patients on the initial blood cultures performed upon admission to the hospital in all included case reports [21-30].

Treatment

Both medical and surgical methods were used to treat IE. All 11 patients received medical treatment in the form of antibiotics. Medical treatment can be divided into empirical and definite management, i.e., antibiotics given once the results of culture and sensitivity are known. Antibiotics used for empirical treatment were not mentioned for the two patients. Of the remaining nine patients, six received cephalosporins, eight received vancomycin, one received metronidazole, one received aztreonam, and one was given clindamycin. Out of the cephalosporins, three patients were given ceftriaxone, and three were given cefepime. One of the patients initially received ceftazidime, which was later changed to ceftriaxone. After the culture and sensitivity results, four patients were given penicillins; one received gentamicin, three received vancomycin, and four received cephalosporins. Amongst the patients who received penicillins, three were given nafcillin, and one was given flucloxacillin. Among the patients treated with cephalosporins, one received ceftriaxone, and three received cefazolin. Changes in antibiotics, if any, were not mentioned for three patients.

**Figure 1:**
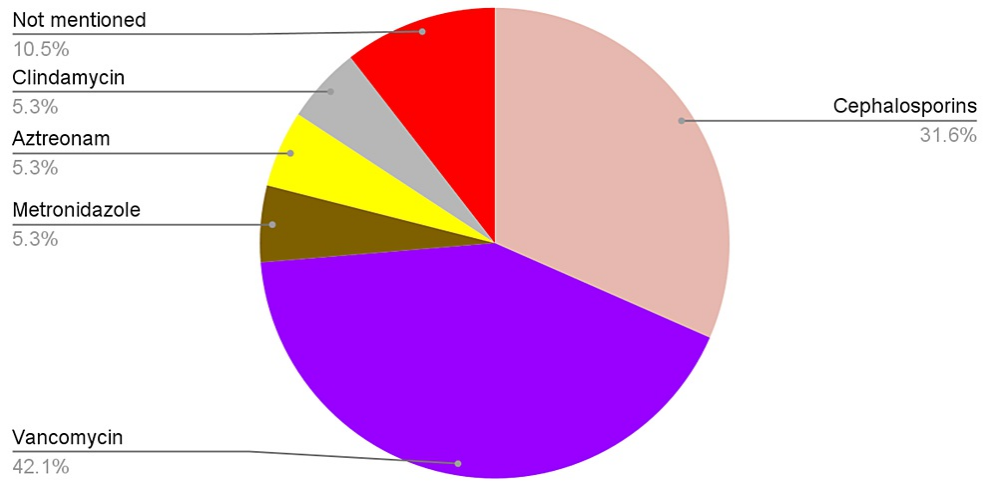
Antibiotics used in empirical management

**Figure 2:**
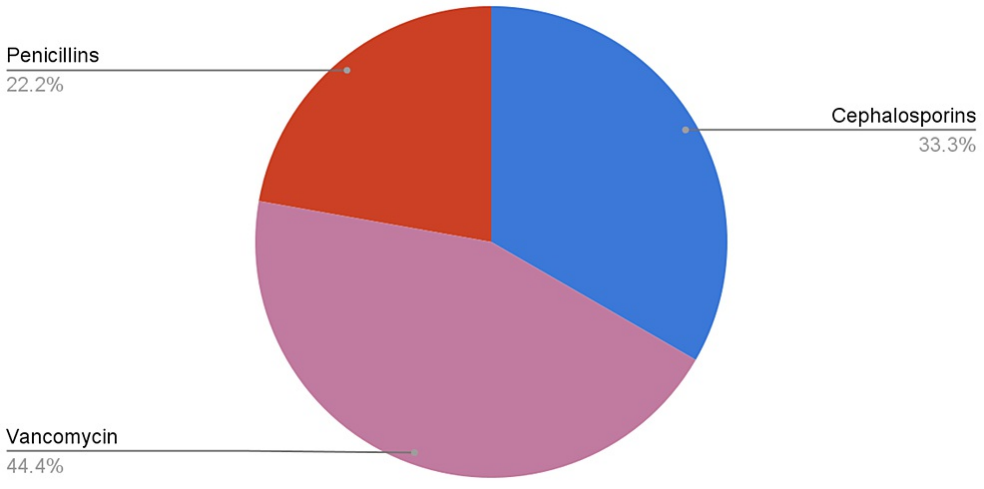
Antibiotics used in definitive management

Figures 1, 2 demonstrate the antibiotic coverage analyzed from the records included in our review.

Of the 11 cases reviewed, eight patients received surgical interventions apart from medical management for IE. Two of the surgical candidates had undergone prior valvular interventions, one patient had prior pacemaker implantation, and one patient had undergone penile implantation surgery. One patient who had valvular implantation previously was deemed a poor surgical candidate due to age and comorbidities and thus received comfort care. Four patients underwent valve replacements: one underwent native aortic valve replacement, one underwent mitral valve replacement, and two underwent aortic valve replacement after previously implanted valves were explanted. The patient who underwent native aortic valve replacement also had other procedures done simultaneously, like ascending autoplasty, aortic root abscess repair, and patch closure. Concomitant coronary artery disease was rectified with coronary artery bypass grafting. Three patients had valve repair; two received tricuspid, and one received mitral valve repair. Tricuspid valve repair included annuloplasty and replacement of septal leaflet with pericardium. Vegetation debulking using the AngioVac system was done for one patient after being classified as a poor surgical candidate. One patient underwent pacemaker explantation after the implanted device was regarded as the source of infection.

Sites Involved

Five different sites were identified to be the sites of IE: the aortic valve, tricuspid valve, mitral valve, papillary muscles, and an atrial pacer lead. The aortic valve was involved in five of the patients [21,22,26,28], tricuspid in three [25,27,30], and mitral in three [23,24,27]. Papillary muscle was affected in one of the patients [27], and atrial pacer lead was the site of vegetation in one patient [29].

Outcomes

Despite aggressive management, two patients died as their condition worsened [21,28]. However, 63.6% of the patients were discharged once their symptoms had been successfully resolved [22-27,29]. Subsequently, one of the discharged patients passed away due to an unknown cause [24]. Post-procedure worsening of tricuspid regurgitation was noted in one patient [30]. Due to a poor prognosis, comfort measures were requested for less than 1% of the patients [26].

*Overall When Were TTE and TEE* *Done?*

After reviewing the case reports, we noted that overall, the TTE was performed first [21-30], since it often provides enough information to make the diagnoses, does not require anesthesia or sedation, is a less invasive procedure, and carries less risk of complications [31,32]. Out of the 10 cases reviewed, only four cases [21,27,28,30] provided information regarding the exact time when the TEE was performed, indicating a lack of consistency in documenting this important detail.

We can confidently say that the most significant imaging modality for diagnosing IE and its consequences is echocardiography (TTE and TEE) [8]. In all cases of suspected endocarditis, TTE should be performed. When TTE is negative, TEE plays a vital role in establishing the presence of IE. It is used to classify lesions and identify local consequences. TEE has a high sensitivity for detecting endocarditis in patients with prosthetic valves, with a sensitivity of more than 90% [33].

Discussion

Summary

Our review included 10 case reports with 11 patients in total. Eight MSSA and three MRSA infections were noted from all the records included in our literature review. Eleven patients with IE were treated with antibiotics, six receiving cephalosporins, eight receiving vancomycin, and four receiving penicillins. Eight patients also underwent surgical interventions; four underwent valve replacements, three had valve repairs, and one underwent vegetation debulking. The aortic valve was involved in five patients, tricuspid in three, and mitral in three. One patient had papillary muscle involvement, and another had vegetation on an atrial pacer lead. One patient was not a surgical candidate due to age and comorbidities and received comfort care. One patient underwent a pacemaker explantation due to infection.

*TTE* *vs. TEE*

An echocardiogram is an imaging technique that works on the principles of sound waves to produce images of heart walls. The probe sends sound waves, which are reflected from heart walls and are picked up by the receiver [34]. TTE is a non-invasive test in which the probe is placed on the patient's chest wall and an echocardiogram is performed, for which no sedation is required. It is an easier and cheaper test to perform. Its disadvantages include operator dependency and low sensitivity [35]. TEE is a semi-invasive procedure in which a probe is passed down the esophagus of the patient, for which mild sedation is required. It produces great-quality images and has higher sensitivity than TTE, but it can cause injuries to the oropharynx, esophagus, and stomach [36]. This is illustrated in Table 4 [37].

**Table 4:** Comparing TTE with TEE TTE: transthoracic echocardiogram; TEE: transesophageal echocardiogram.

	TTE	TEE
Invasiveness	Non-invasive	Semi-invasive
Advantages	Easy to perform, cheaper, better safety profile	Higher image resolution, higher sensitivity
Disadvantages	Image affected by obesity, hyperinflated lungs, chest deformity, shadowing of prosthetic valves may affect the results	May cause injuries to the oropharynx, esophagus, and stomach

When diagnosing MRSA or MSSA endocarditis, both TEE and TTE are important diagnostic tools. TTE is typically used as the first diagnostic test with a moderate sensitivity of 75% and a specificity close to 100% [37].TEE is more effective in detecting vascular vegetation and identifying cardiac complications such as abscesses or intracardiac fistula, with a sensitivity of over 90% [38]. Nevertheless, specificity is not 100% and false-positive results are possible due to cardiac tumors, mural thrombi, or fibrous strands [1]. In terms of safety, even though TEE is an invasive test that requires conscious sedation, studies have shown that TEE is associated with a low risk of complications, evidenced by a case series by Poelaert et al. (1995) of 108 patients who underwent TEE in the ICU with a complication rate of 1.6% for esophageal injury or bleeding [39]. In terms of practicality, TEE requires specialized equipment and expertise and may take longer to perform; on the other hand, TEE is a non-invasive test that can be performed at the bedside [40].

In our case series, vegetations were identified with TEE in all of the patients; one patient had a negative result on the initial echocardiogram but was later diagnosed with a second TEE five days later. On the other hand, TTE reported vegetation in two out of the 11 cases reviewed. These findings highlight the superior sensitivity of TEE when compared to TTE in the diagnosis of IE.

Other Diagnostics

Even though both TTE and TEE are a cornerstone in diagnosing IE, other diagnostic modalities listed by the European Society of Cardiology for diagnosis of prosthetic valve endocarditis (PVE) include multi-slice CT, contrast-enhanced multi-slice CT, and 18F-fluorodeoxyglucose positron emission tomography-CT (18F-FDG PET/CT) after administration of 18F-fluorodeoxyglucose (18F-FDG) [26].

18F-FDG PET/CT should be considered in all patients with suspected IE who have an inconclusive or negative TEE [26]. It also determines clinically important extracardiac foci of infection and malignancy leading to notable effects in treatment strategies [41].

With the implementation of computed tomography angiography (CTA), it became easier to get very detailed morphology and systemic IE-associated pathology, which is used in difficult-to-diagnose IE and complicated PVE [42].

When CTA is combined with 18F-FDG PET-CT in the early stage of the disease, it is seen to potentially increase diagnostic yield [42].

In addition to this, radiolabeled leukocyte scintigraphy is an emerging diagnostic whole-body test useful in early PVE [42].

Treatment

Prior to the discovery of antibiotics, IE was always untreatable and fatal [43]. A multidisciplinary team should oversee the management of IE, which should include cardiologists, specialists in infectious diseases and/or microbiologists, cardiac surgeons, nephrologists, neurologists, and radiologists [2,43]. Bactericidal antibiotic regimens, frequently in combination, are required for effective microbiological clearance [2,8]. The specific treatment will depend on the severity of the infection and the type of microorganism causing it. Therapy with antibiotics is frequently empirical and depends on patient and epidemiological criteria. The etiology varies by geography, reflecting changes in local pathogens, antibiotic use prior to obtaining blood cultures, and diagnostic tests [8,34]. Antibiotic therapy is typically administered for six (up to eight) weeks in non-critically sick patients with IE [44]. The presence or lack of antibiotic resistance influences antimicrobial selections for *Staphylococcus aureus* IE. Because anti-staphylococcal beta-lactam drugs, such as nafcillin, are associated with greater cure rates for MSSA bacteremia than vancomycin, they are indicated in the therapy of MSSA. Cefazolin can be used instead of nafcillin to treat individuals who have a non-anaphylactic penicillin allergy. Vancomycin is the antibiotic of choice for MRSA and a viable option for patients who cannot tolerate therapy with penicillin or ceftriaxone [11,34]. Daptomycin is a suitable option for its effectiveness against gram-positive bacteria, mainly *S. aureus* and MRSA, but also against beta-hemolytic streptococci; nevertheless, careful dosing is required [11,44]. In patients with severe valvular dysfunction, heart failure, paravalvular abscess, septic embolization, large vegetation (>10 mm on left-sided valves), infection refractory to medical therapy, and fungal IE, surgery is recommended. Surgery usually done for IE requires a sternotomy. Generally, surgery is done to debride necrotic and infectious tissues [44-46]. The choice of valve repair and replacement depends on the type of valve involved and the severity of the infection [45]. Valve repair is generally preferred for the mitral valve. For the aortic valve, valve replacement is usually done [45]. Age, past medical history, and compliance with anticoagulation should be considered when choosing the type of valve (mechanical vs. tissue). Allograft is associated with a low risk of IE for aortic root replacement in both native and prosthetic aortic valve endocarditis [45].

Antibiotic therapy for four to six weeks is generally recommended after surgery [46]. Of patients with IE, 30% develop complications within the first 12 months, requiring surgery. Therefore, patients should be followed up very closely in the first year. Appropriate education and counseling regarding risk factors, and education about endocarditis prophylaxis should be given [47].

MRSA Endocarditis vs. MSSA

Endocarditis is an infection of the inner lining of the heart and occurs when microorganisms enter the bloodstream and then travel to the heart. It can be caused by various bacteria, including MRSA and MSSA. MRSA is a type of *Staphylococcus aureus* bacteria resistant to many antibiotics, including methicillin, which is commonly used to treat *Staphylococcus* infection. MSSA, on the other hand, is a type of *Staphylococcus aureus* bacteria that is sensitive to methicillin and can be treated with a broader range of antibiotics, including penicillin [48].

Endocarditis caused by MRSA and MSSA can have similar symptoms, including fever, fatigue, and heart murmur. However, MRSA endocarditis may be more difficult to treat due to its resistance to beta-lactamase antibiotics. Treatment typically involves a combination of antibiotics and sometimes surgical intervention to remove infected tissue or repair damaged heart valves [48].

It is worth noting that MRSA endocarditis is typically associated with higher mortality when compared to MSSA endocarditis and may require more aggressive treatment [48]. It is important to seek prompt medical attention if you suspect you may have endocarditis, regardless of the cause, as early treatment can improve outcomes.

Limitations

The primary study limitations are inherent to the narrative review study design. Pooling of different case reports with differences in age, gender, clinical presentations, comorbidities, in-patient stay, treatment modalities, and outcome introduces imprecision in the results because of heterogeneity. Also, the presence of previous endocarditis events may have been underestimated in the study sample because this was obtained by a review of available reports. Data on when the TTE was done was not available in most of the case reports. Since TEE is central in diagnosing IE, it is relevant to know when TEE was done, thereby increasing the likelihood of compensating accurate data analysis. We also did not have direct patient‐level information on antibiotic susceptibility or how this changed over time. Moreover, there has been the exclusion of case reports with patients who are immunocompromised, pregnant ladies, and pediatric age groups in our study.

## Conclusions

IE is an uncommon but potentially deadly infection, and patients with this disease should be evaluated preferentially by a multidisciplinary approach. Early involvement of physicians from different specialties and creating a team for early diagnosis and effective management can prevent morbidity and mortality. Early initiation of appropriate antibiotic therapy is essential in improving clinical outcomes. But due to the emerging antibiotic resistance, *Staphylococcus aureus* bacteremia can remain aggressive. It seems reasonable to conclude that possible new synergistic combinations of antibiotics should be used.

Also, due to its varying clinical presentations, a high index of clinical suspicion is needed to diagnose this disease early. All adult patients with *S. aureus* bacteremia should undergo echocardiography. TTE and TEE are of utmost value in the diagnosis of IE. The use of TEE is especially responsible for high accuracy and specificity in diagnosing and assessing complications. The additional value of TEE when TTE is negative or inconclusive is well defined in the strong suspicion of IE or the presence of valvular prostheses. The recommendations of TEE will vary on an individual patient basis, the patient's clinical status, the possible limitations, and the suspected new complications. However, new studies need to authenticate the indication of TEE as a primary examination.
